# Repeat prenatal corticosteroid prior to preterm birth: a systematic review and individual participant data meta-analysis for the PRECISE study group (prenatal repeat corticosteroid international IPD study group: assessing the effects using the best level of evidence) - study protocol

**DOI:** 10.1186/2046-4053-1-12

**Published:** 2012-02-12

**Authors:** Caroline A Crowther, Fariba Aghajafari, Lisa M Askie, Elizabeth V Asztalos, Peter Brocklehurst, Tanya K Bubner, Lex W Doyle, Sourabh Dutta, Thomas J Garite, Debra A Guinn, Mikko Hallman, Mary E Hannah, Pollyanna Hardy, Kimberly Maurel, Premasish Mazumder, Cindy McEvoy, Philippa F Middleton, Kellie E Murphy, Outi M Peltoniemi, Dawn Peters, Lisa Sullivan, Elizabeth A Thom, Merryn Voysey, Ronald J Wapner, Lisa Yelland, Sasha Zhang

**Affiliations:** 1 Australian Research Centre for Health of Women and Babies (ARCH), Discipline of Obstetrics and Gynaecology, The University of Adelaide, Women's and Children's Hospital, Adelaide, Australia; 2 Faculty of Medicine, University of Calgary, Calgary, Canada; 3 National Health and Medical Research Council Clinical Trials Centre, University of Sydney, Sydney, Australia; 4 Department of Paediatrics and Obstetrics/Gynaecology, University of Toronto, Toronto, Canada; 5 National Perinatal Epidemiology Unit, University of Oxford, Oxford, UK; 6 Department of Obstetrics and Gynaecology, University of Melbourne, Melbourne, Australia; 7 Department of Neonatology, Postgraduate Institute of Medical Education and Research, Chandigarh, India; 8 Pediatrix Medical Group, Sunrise, USA; 9 Northwest Perinatal Centre, Portland, USA; 10 Department of Paediatrics, University of Oulu, Oulu, Finland; 11 Department of Obstetrics and Gynaecology, University of Toronto, Toronto, Canada; 12 Department of Pediatrics, Postgraduate Institute of Medical Education and Research, Chandigarh, India; 13 Department of Pediatrics, Oregon Health and Science University, Portland, USA; 14 Public Health and Preventive Medicine, Oregon Health and Science University, Portland, USA; 15 School of Public Health, Boston University, Boston, USA; 16 The Biostatistics Centre, George Washington University, Washington DC, USA; 17 Centre for Statistics in Medicine, University of Oxford, Oxford, UK; 18 Department of Obstetrics and Gynecology, Division of Maternal Fetal Medicine, Columbia University Medical Centre, New York, USA

## Abstract

**Background:**

The aim of this individual participant data (IPD) meta-analysis is to assess whether the effects of repeat prenatal corticosteroid treatment given to women at risk of preterm birth to benefit their babies are modified in a clinically meaningful way by factors related to the women or the trial protocol.

**Methods/Design:**

The Prenatal Repeat Corticosteroid International IPD Study Group: assessing the effects using the best level of Evidence (PRECISE) Group will conduct an IPD meta-analysis. The PRECISE International Collaborative Group was formed in 2010 and data collection commenced in 2011. Eleven trials with up to 5,000 women and 6,000 infants are eligible for the PRECISE IPD meta-analysis. The primary study outcomes for the infants will be serious neonatal outcome (defined by the PRECISE International IPD Study Group as one of death (foetal, neonatal or infant); severe respiratory disease; severe intraventricular haemorrhage (grade 3 and 4); chronic lung disease; necrotising enterocolitis; serious retinopathy of prematurity; and cystic periventricular leukomalacia); use of respiratory support (defined as mechanical ventilation or continuous positive airways pressure or other respiratory support); and birth weight (Z-scores). For the children, the primary study outcomes will be death or any neurological disability (however defined by trialists at childhood follow up and may include developmental delay or intellectual impairment (developmental quotient or intelligence quotient more than one standard deviation below the mean), cerebral palsy (abnormality of tone with motor dysfunction), blindness (for example, corrected visual acuity worse than 6/60 in the better eye) or deafness (for example, hearing loss requiring amplification or worse)). For the women, the primary outcome will be maternal sepsis (defined as chorioamnionitis; pyrexia after trial entry requiring the use of antibiotics; puerperal sepsis; intrapartum fever requiring the use of antibiotics; or postnatal pyrexia).

**Discussion:**

Data analyses are expected to commence in 2011 with results publicly available in 2012.

## Background

### Clinical significance of respiratory distress syndrome and other neonatal morbidities in preterm birth

Respiratory distress syndrome, as a consequence of immature lung development, is a complication of preterm birth and the major cause of early neonatal mortality and morbidity [1,2]. Infants born very preterm (less than 32 weeks' gestation) often require respiratory support, with significant numbers requiring assisted ventilation, and 9.4% remain dependent on oxygen therapy 28 days after birth and are diagnosed with chronic lung disease [1]. Of infants born very preterm admitted for neonatal intensive care, a substantial proportion (21.6%) will have an intraventricular haemorrhage (IVH) with 5.8% being Grade 3 or 4 IVH; 1.7% will have cystic periventricular leukomalacia; and 4.1% of babies will have severe retinopathy of prematurity [1]. Children born preterm who survive have an increased risk of re-hospitalisation after discharge home and a recognised higher risk of subsequent long-term neurodevelopmental impairments, including cerebral palsy [3,4], than do children born at term. The personal and emotional costs for affected individuals and their families are high, as are the immediate and long-term monetary costs of these morbidities for parents and society [3-6].

Strategies to reduce the risk of neonatal respiratory disease for preterm birth continue to receive considerable attention [7-9]. A single course of prenatal corticosteroids compared with placebo has not been shown to be effective in babies who are born more than seven days after treatment [7]. Specifically, there is insufficient evidence of a reduction in the incidence of respiratory distress syndrome or neonatal mortality [7,10,11] and birth weight is significantly reduced [7]. This evidence led to the suggestion [12] and uptake into clinical practice within Australia [13] and other countries [14], with minimal formal assessment, of repeating the dose of prenatal corticosteroids in the 34% to 40% [7,10] of women who remained at risk of preterm birth seven or more days after the initial course.

Observational studies, with their inherent risk of bias, have given conflicting results, some suggesting adverse effects of repeat corticosteroids on measures of foetal growth [15] and on delayed development in early childhood [16], whilst others have indicated a possible reduction in cerebral palsy [17]. Given the need for better quality evidence about the benefits and harms of repeat prenatal corticosteroids, randomised clinical trials have now been reported [18-27].

A recent Cochrane systematic review of published aggregate data was unable to answer some important clinical questions which this individual participant data (IPD) meta-analysis will address [28].

#### Summary of the Cochrane systematic review of the aggregate data in 2011

It remains unclear whether repeat dose(s) of prenatal corticosteroids are beneficial. The most recent Cochrane systematic review that assesses the use of repeat prenatal corticosteroids for women at risk of preterm birth to prevent neonatal respiratory disease included ten trials (over 4,730 women and 5,650 babies) with low to moderate bias [28].

Five of the trials were conducted in the US [20,21,23,26,27]; one in each of Canada [18], India [22] and Finland [25]; one in Australia and New Zealand [19]; and one involved 20 countries [24].

Six trials [18,19,21-23,26] gave repeat corticosteroids at seven day intervals if risk of preterm birth remained, one trial [24] at 14 day intervals and three trials [20,25,27] specifically targeted women for 'rescue therapy' (repeat doses only given when preterm birth was considered imminent).

There was diversity in the inclusion and exclusion criteria for the ten included trials, with wide variation in the reasons women were at risk of preterm birth (preterm labour, preterm prelabour rupture of the membranes, antepartum haemorrhage, pre-eclampsia, foetal growth restriction, cervical incompetence and multiple pregnancy); the gestational age women were eligible (from 24 to 34 weeks); and the time of treatment prior to expected preterm birth. All women received a single course of prenatal corticosteroids one week or more before trial entry. However, the type, amount and timing regimen for administration of the corticosteroid given before the trial varied between trials.

Treatment of women who remain at risk of preterm birth seven or more days after an initial course of prenatal corticosteroids with repeat dose(s), compared with no repeat corticosteroid treatment, reduced the risk of their infants being affected by the primary outcomes, respiratory distress syndrome (risk ratio (RR) 0.83, 95% confidence interval (CI) 0.75 to 0.91; eight trials; 3,206 infants; numbers needed to treat (NNT) 17, 95% CI 11 to 32) and serious infant outcome (RR 0.84, 95% CI 0.75 to 0.94; seven trials; 5,094 infants; NNT 30, 95% CI 19 to 79).

Treatment with repeat dose(s) of corticosteroid was associated with a reduction in mean birth weight (mean difference -75.8 g, 95% CI -117.6 g to -34 g; nine trials; 5,626 infants). However, outcomes that adjusted birth weight for gestational age (birth weight Z-scores, birth weight multiples of the median and small for gestational age) did not differ between treatment groups.

At early childhood follow-up, no statistically significant differences were seen for infants exposed to repeat prenatal corticosteroids compared with unexposed infants for the primary outcomes, total deaths (RR 0.98, 95% CI 0.72 to 1.33; two trials; 3,250 infants), survival free of any disability (RR 1.00, 95% CI 0.97 to 1.04; two trials; 3,164 infants), survival free of major disability (RR 0.77, 95% CI 0.55 to 1.08; one trial; 999 infants), disability (RR 0.98, 95% CI 0.83 to 1.16; one trial; 999 infants) or serious outcome (RR 0.99, 95% CI 0.87 to 1.12; two trials; 3164 infants), or in the secondary outcome growth assessments [28].

For maximising benefit and minimising harm, many questions remain. How can the potentially important benefits observed be applied to individual women who have different reasons for, and levels of risk, of preterm birth? If repeat prenatal corticosteroids are to be recommended, what are the optimal gestational ages for administration, the optimal number of repeat treatments that should be given, and at what dose and timing? Individual participant meta-analysis of the data from the 11 trials now available may help to answer these questions.

#### Overcoming limitations: conducting an individual participant data meta-analysis

Analysis of thoroughly checked and updated data from individual participants in all the available randomised trials has been described as the gold standard in systematic reviews [29]. Estimates of treatment effects are often different from those obtained from aggregate published data due to inclusion of additional or updated data. The methods and advantages of IPD review have been well described [30,31].

An integral component of conducting an IPD meta-analysis is the formation of an international collaborative group of trialists where all researchers endorse the IPD protocol and provide data from their trials. This generates additional benefits that include more complete identification of trials and of trial details; compliance with standard definitions; provision of missing data on characteristics of trials, all women who were randomised and their babies, and outcomes; more balanced interpretation, endorsement and global dissemination of results; and better clarification and consensus on future research needed with the opportunity for on-going international collaborations [32].

### Objectives

Our aim is to assess, using IPD meta-analyses, the effects on important clinical outcomes of repeat prenatal corticosteroid treatment given to women at risk of preterm birth to benefit their babies, both short-term and long-term, and whether treatment effects differ in a clinically meaningful way between important pre-specified participant-level and trial-level characteristics.

### Research Questions

For maximising benefit and minimising harm, the main research questions to be addressed in this review are:

1. Are repeat prenatal corticosteroids more effective in some women by reason of their risk of preterm birth?

2. If the use of repeat prenatal corticosteroids is recommended, what is the best gestational age to maximise benefit?

3. What dose, number of repeat doses and timing prior to birth is optimal?

4. What is the minimal effective and safe dose of repeat prenatal corticosteroids?

5. Is a single rescue steroid dose effective?

## Methods/Design

### Study design

IPD meta-analysis.

### Inclusion and exclusion criteria for the studies

Studies, published or unpublished, will be included if they were randomised trials with adequate allocation concealment and report data on one or more of the pre-stated outcomes. Quasi-random study designs will not be included. As of September 2011, 11 trials with up to 5,000 women and 6,000 infants are eligible for the Prenatal Repeat Corticosteroid International IPD Study Group: assessing the effects using the best level of Evidence (PRECISE) IPD meta-analysis [18-27,33] (Table 1).

**Table 1:** Eligible trials with their inclusion criteria and corticosteroid regimens as at September 2011

TRIAL	Gestational age at entry (weeks)	Repeat corticosteroid timing	Repeat corticosteroid regimen	Number of repeat courses
**Garite 2009 **[20]	25 to < 33	Birth expected < 7 days	2 × 12 mg betamethasone 24 hours apart; or 4 × 6 mg dexamethasone 12 hours apart	1
**Peltoniemi 2007 **[25]	< 34	Birth expected < 48 hours	1 × 12 mg betamethasone	1
**Aghajafari 2002 **[18]	24 to 30	Weekly until 33 weeks, or birth if still at risk of preterm birth	2 × 12 mg betamethasone 24 hours apart	varied
**Crowther 2006 **[19]	< 32	Weekly until < 32 weeks, and if still at risk of preterm birth	1 × 11.4 mg betamethasone	varied
**Guinn 2001 **[21]	24 to < 33	Weekly until 34 weeks	2 × 12 mg betamethasone 24 hours apart	varied
**Mazumder 2008 **[22]	26 to 33	Weekly until < 34 weeks	2 × 12 mg betamethasone 24 hours apart	varied
**McEvoy 2002 **[23]	25 to 33	Weekly until 34 weeks	2 × 12 mg betamethasone 24 hours apart	varied
**McEvoy 2010 **[27]	26 to 34	Rescue course 14 days after initial dose; < 34 weeks	2 × 12 mg betamethasone 24 hours apart	1
**Murphy 2006 **[24]	25 to 32	Fortnightly until 33 weeks	2 × 12 mg betamethasone 24 hours apart	varied
**Wapner 2006 **[26]	23 to < 32	Weekly until < 34 weeks	2 × 12 mg betamethasone; 24 hours apart	varied
**Brocklehurst **[14]	< 32	Weekly (with 10 and 14 day intervals being used in some centres)	2 × 12 mg betamethasone; 12 or 24 hours apart.	varied

Eligibility of trials will be assessed independently and unblinded for author and journal by two members of the PRECISE IPD Project Team. Any differences in opinion regarding eligibility will be resolved by discussion. If IPD are unavailable for any eligible trial, the trial will still be included in the review and aggregate data will be used for sensitivity analyses wherever possible.

### Types of participants

Women considered at risk of preterm birth (< 37 weeks gestation) who have already received a single course of prenatal corticosteroid seven or more days previously.

### Types of interventions

Corticosteroid administered to the women intravenously, intramuscularly or orally, compared with either placebo or no placebo. Trials in which the foetus received corticosteroids directly will not be included.

### Search strategy to identify potential trials

We will use the search strategy developed by the Cochrane Pregnancy and Childbirth Review Group for the Cochrane review, which identifies trials from quarterly searches of the Cochrane Central Register of Controlled Trials (CENTRAL); weekly searches of MEDLINE; hand searches of 30 journals and the proceedings of major conferences; weekly current awareness alerts for a further 44 journals plus monthly BioMed Central email alerts.

We will also search CENTRAL, MEDLINE and EMBASE to identify trials published since the search cut-off date of 31 August 2008 for the Cochrane review; using the terms [repeat or multiple] and [antenatal or prenatal] and [corticosteroid* or steroid* or glucocorticoid* or betamethason* or dexamethason* or hydrocortison*].

In addition we will access Current Controlled Trials http://www.controlled-trials.com and the Australian and New Zealand Trials Register http://www.anzctr.org.au to identify recently completed or on-going trials.

Experts in the field and trialists will be asked if they know of any unpublished or other trials.

#### Data collection and management

Data collected will include all women randomised and coded for anonymity (date of birth, centre identification); baseline data for descriptive purposes and analyses (reason at risk of preterm birth, gestational age at trial entry, plurality of the pregnancy, expected date of birth); details of the intervention given (date of randomisation, allocated intervention, type and dose of corticosteroid given, interval between treatments, whether re-treatment given and amount); and outcomes, to allow planned analyses.

Trialists will provide de-identified IPD in any convenient format by encrypted, electronic transfer where possible or other means as needed. The individual trial data will be recoded as required and stored in a custom designed secure database which will only be accessible by authorised personnel of the PRECISE Data Management Group. Trialists will be asked to verify all recoded data prior to any analysis and the data will not be used for any other purpose without permission of all collaborators.

The data will be checked with respect to range, internal consistency, missing or extreme values, errors and consistency with published reports. Trial details, such as randomisation methods, and intervention details will be cross-checked against published reports, trial protocols and data collection sheets. Inconsistencies or missing data will be discussed with the individual trialists and attempts will be made to resolve any problems by consensus. Each trial will be checked individually and the trial data will be sent to the trialists for verification.

#### Data items to be collected

##### Trial level information

1. Dates the trial opened and closed accrual

2. Number of participants randomised

3. Informed consent procedures

4. Methods of random allocation

5. Stratification factors

6. Methods of allocation concealment

7. Blinding procedures for outcome assessment

8. Purpose repeat corticosteroid treatment given (prophylaxis against preterm birth; 'rescue therapy' when preterm birth is imminent; other)

9. Details of the planned intervention in the experimental arms (drug frequency; timing; doses):

a. Type of repeat prenatal corticosteroid treatment given

b. Number of repeat doses planned to be given

c. Minimum planned interval between the initial corticosteroid and the repeat dose

d. Minimum planned interval between repeat steroid treatments

e. Planned dose of corticosteroid to be given per repeat treatment

f. Planned dose of repeat steroid drug exposure per week

g. Total drug exposure planned

10. Details of the planned intervention in the control arm

##### Participant-level information - maternal characteristics at study entry

1. Unique identification coded for anonymity

2. Maternal age

3. Body mass index

4. Parity

5. Ethnicity

6. Public or private patient

7. Previous obstetric history

8. Reason the woman was considered to be at risk of preterm birth (preterm labour; presence or absence of ruptured membranes; antepartum haemorrhage; pre-eclampsia or eclampsia; foetal growth restriction; suspected foetal jeopardy; cervical incompetence; maternal disease; multiple pregnancy)

9. Pre-trial treatment with corticosteroids (gestation dose given; corticosteroids used; dose regimen)

10. Reason repeat prenatal corticosteroid treatment was considered (prophylaxis against preterm birth; 'rescue therapy' when preterm birth is imminent; other)

11. Number of foetuses *in utero*

##### Participant-level information - maternal information after trial entry

1. Gestational age when repeat prenatal corticosteroid treatment was started

2. Time prior to birth repeat prenatal corticosteroid treatment was given

3. Adverse events for the woman at time of treatment

4. Antenatal information after trial entry

5. Intrapartum information

6. Postnatal information

##### Participant-level information - data on actual study intervention relating to regimens

1. Type of repeat prenatal corticosteroid treatment given

2. Number of repeat doses actually given

3. Minimum actual interval between the initial corticosteroid and the study repeat dose

4. Minimum actual interval between repeat steroid treatments

5. Actual dose of corticosteroid given per repeat treatment

6. Actual dose of repeat steroid drug exposure per week

7. Total actual drug exposure

8. Actual rescue treatment versus weekly treatment

##### Participant-level information - neonatal information

1. Unique baby identification and mother identification coded for anonymity

2. Date and time of birth

3. Gestational age at birth

4. Gender

5. Mode of birth

6. Birth weight, length, head circumference

7. Apgar score at 1 and 5 minutes

8. Neonatal complications and/or status

9. Mortality and age at death

10. Cause of death

11. Childhood follow-up assessments

#### Planned analyses

A detailed statistical analysis plan will be prepared by the PRECISE Data Management Group and agreed upon by the PRECISE IPD Project Team and the PRECISE International IPD Study Group prior to the data analyses. Any analyses conducted will be based on the checked and updated individual participant data from all available trials. All randomised participants with outcome data available will be included in the analyses, which will be performed on an intention to treat basis, according to the treatment allocation at randomisation.

For each of the outcomes, a one-stage approach to analysis will be taken so that the IPD from all eligible trials are included in a single model [34]. Fitting a single model for each outcome variable will enable the variation across trials to be accounted for within the model by including a fixed trial effect. A treatment by trial interaction term will be tested to assess heterogeneity of treatment effect across trials. If excessive statistical heterogeneity in treatment effect or inconsistency across trials is detected (that is, if the trial by treatment interaction term is significant), then the rationale for combining trials will be questioned and the source of heterogeneity explored.

Binary outcomes will be analysed using log binomial regression models and results will be presented as risk ratios with 95% CI and associated two-sided *P *values. Continuous outcomes will be analysed using linear regression models and results will be presented as differences in means with 95% CI and two-sided *P *values. Correlation between outcomes due to multiple births will be taken into account using generalised estimating equations as appropriate.

Any differences in treatment effect between pre-specified subgroups will be assessed by testing a treatment by subgroup interaction term within the model [35].

#### Outcomes

Outcomes have been chosen to be most representative of the clinically important measures of effectiveness and safety, including serious outcomes, for the women and their babies. The definitions given by the trialists are used unless otherwise stated.

#### Primary outcomes

##### For the infants

1. serious outcome, defined by the PRECISE International IPD Study Group as one of death (foetal, neonatal or infant), severe respiratory disease, severe IVH (grade 3 and 4), chronic lung disease (oxygen dependent at 36 weeks postnatal age), definite necrotising enterocolitis, severe retinopathy of prematurity (Stage 3 or worse in the better eye) or cystic periventricular leukomalacia

2. use of respiratory support, defined as mechanical ventilation or continuous positive airways pressure or other respiratory support

3. birth weight (Z-scores) [36].

##### For the children

1. death or any neurosensory disability at childhood follow up, and may include developmental delay or intellectual impairment (developmental quotient or intelligence quotient more than one standard deviation below the mean), cerebral palsy (abnormality of tone with motor dysfunction), blindness (for example, corrected visual acuity worse than 6/60 in the better eye) or deafness (for example, hearing loss requiring amplification or worse).

##### For the women

1. maternal sepsis, defined as chorioamnionitis during labour, pyrexia after trial entry requiring the use of antibiotics, puerperal sepsis, intrapartum fever requiring the use of antibiotics or postnatal pyrexia.

#### Secondary outcomes

##### For the infants

1. foetal, neonatal or later death (up to primary hospital discharge)

2. severe respiratory disease

3. severe IVH (grade 3 and 4)

4. chronic lung disease

5. necrotising enterocolitis

6. retinopathy of prematurity

7. cystic periventricular leukomalacia

8. respiratory distress syndrome

9. IVH

10. patent ductus arteriosus

11. neonatal encephalopathy

12. serious outcome (may include: foetal, neonatal or later death; respiratory distress syndrome; severe lung disease; chronic lung disease; intraventricular haemorrhage; patent ductus arteriosus; neonatal encephalopathy; retinopathy of prematurity)

13. gestational age at birth (actual gestational age, preterm birth less than 37 weeks, very preterm birth less than 32 weeks and extremely preterm birth less than 28 weeks)

14. interval between trial entry and birth

15. small-for-gestational age [36]

16. birth weight (raw values)

17. head circumference at birth (raw values and Z-scores) [36]

18. length at birth (raw values and Z-scores) [36]

19. placental weight

20. Apgar score less than seven at five minutes

21. resuscitation at birth

22. duration of respiratory support

23. use of oxygen supplementation

24. duration of oxygen supplementation

25. use of surfactant

26. air leak syndrome

27. pulmonary hypertension

28. use of inotropic support

29. use of nitric oxide for respiratory support

30. early neonatal infection

31. proven neonatal infection while in the neonatal intensive care unit

32. use of postnatal corticosteroids

33. neonatal blood pressure (systolic, diastolic and mean arterial blood pressure)

34. growth assessments at primary hospital discharge (Z-scores for weight, head circumference, length) [36]

35. hypothalamic/pituitary/adrenal axis suppression.

##### For the children (follow up)

Categories will be collapsed and/or combined where data are insufficient:

1. death (foetal, neonatal or later death up to the time of follow up)

2. neurosensory impairments

a. cerebral palsy (categorised as nil, mild, moderate or severe)

b. developmental delay or intellectual impairment (categorised as nil, mild (< 85), moderate (< 70) or severe (< 55))

c. blindness

d. deafness

e. gross motor dysfunction, defined as mild, moderate or severe, by trialists or by the Gross Motor Classification System (score 1 to 5), if available

f. motor delay (categorised as nil, mild (< 85), moderate (< 70) or severe (< 55) by appropriate mode of assessment)

3. any neurosensory disability, defined as developmental delay or intellectual impairment (developmental quotient or intelligence quotient more than one standard deviation below the mean), cerebral palsy (abnormality of tone with motor dysfunction), blindness or deafness, at follow up later in childhood

4. major neurosensory disability, defined as any moderate or severe neurosensory impairment

5. survival free of major neurosensory disability (alive and without major disability)

6. growth assessments at childhood follow up (Z-scores for weight, head circumference, height) [37]

7. child behaviour

8. child temperament

9. respiratory disease

10. blood pressure.

##### For the women

1. chorioamnionitis during labour

2. pyrexia after trial entry requiring the use of antibiotics

3. puerperal sepsis

4. intrapartum fever requiring the use of antibiotics

5. postnatal pyrexia

6. death

7. preterm prelabour rupture of the membranes after trial entry

8. hypertension

9. mode of birth (normal vaginal birth, operative vaginal birth, Caesarean section)

10. postpartum haemorrhage

11. breastfeeding at hospital discharge

12. postnatal depression

13. adverse effects of corticosteroid therapy (including gastrointestinal upset, glucose intolerance, insomnia, pain at the injection site, bruising at the injection site, infection at injection site, weight gain, Cushing appearance).

##### Use of health services

1. length of antenatal hospitalisation for the women

2. length of postnatal hospitalisation for the women

3. maternal admission to intensive care unit

4. admission to neonatal intensive care unit

5. length of stay in neonatal intensive care unit

6. length of neonatal hospitalisation.

#### Planned subgroup analyses

Where sufficient data exist, subgroup comparisons will be conducted using the five infant, child and maternal primary outcomes, as well as the individual components of each composite outcome. Any differences in treatment effect between subgroups will be assessed by testing a treatment by subgroup interaction term within the model. Some subgroups may be combined if data are insufficient.

#### Subgroups

##### Trial-level characteristics

1. Type of repeat prenatal corticosteroid treatment given (betamethasone or dexamethasone).

2. Number of repeat doses planned to be given.

3. Minimum planned interval between the initial corticosteroid and the first repeat dose.

4. Minimum planned interval between repeat steroid treatments.

5. Planned dose of corticosteroid per repeat treatment.

6. Planned dose of repeat steroid drug exposure per week.

7. Total dose of repeat steroid drug planned.

##### Participant-level characteristics

1. Reason the woman was considered to be at risk of preterm birth (preterm labour, the presence or absence of ruptured membranes, antepartum haemorrhage, pre-eclampsia and/or eclampsia, foetal growth restriction, suspected foetal jeopardy, cervical incompetence, maternal disease and multiple pregnancy).

2. Purpose trial treatment given (prophylaxis against preterm birth; 'rescue therapy' when preterm birth is imminent; other).

3. Number of foetuses *in utero *(singleton versus multiple).

4. Gestational age when first trial treatment was given (< 26; 26 to < 28; 28 to < 30; 30 to < 32; 32 to < 34 completed weeks at randomisation).

5. Time prior to birth last dose of trial treatment was given (< 1; 1 to < 2; 2 to < 4; 4 to < 7; 7 to < 10; 10 to < 14; ≥ 14 days).

6. Actual rescue treatment versus weekly treatment.

#### Additional exposure analyses

1. Number of repeat doses actually given.

2. Minimum actual interval between the initial corticosteroid and the first trial treatment.

3. Minimum actual interval between repeat trial treatments.

4. Actual dose of trial treatment given per repeat treatment.

5. Actual dose of repeat trial treatment exposure per week.

6. Total actual trial treatment exposure.

#### Planned sensitivity analyses

To assess whether the results are robust to trial design and quality, the following sensitivity analyses will be performed on the primary outcomes, as well as the individual components of each composite outcome (unless otherwise specified): exclusion of trials with small sample size (less than 100 study participants); exclusion of trials with high rate of missingness and/or exclusions where the losses are considered to have the potential to impact on the results; inclusion of aggregate data from trials where individual participant data are unavailable; exclusion of trials where not all variables used to define the outcome were collected (composite outcomes only); and random effects models with trial included as a random effect (all primary and secondary outcomes).

#### Multiple comparisons

A large number of outcomes are being investigated in this study, which increases the chance of observing 'false positive' results. However, all outcomes are important in giving a full clinical picture that considers the benefits and risks to both mothers and infants. We do not plan formal statistical adjustment of *P *values to account for multiple comparisons due to the non-independence of outcomes in this study. Results will be interpreted with caution.

#### Ethics and management issues

##### Ethical considerations

Participants in the individual trials have previously given informed consent to participate in their respective trial. The data for this project are to be used for the purpose for which they were originally collected and are available through an agreement between all trialists of the collaborative group. These trialists remain the custodian of their original individual trial data at all times.

#### Project Management

For the purpose of this project, an international Collaborative Group, The PRECISE Group, will manage the project and consists of groups with specific responsibilities and tasks:

##### The PRECISE IPD Project Team

The PRECISE IPD Project Team is the Steering Group which is responsible for the project's management decisions and the daily management of the Collaboration. The Project Team's tasks are to design the project's protocol and analysis plan, organise The PRECISE Group Meetings and act as a liaison between all the members of The PRECISE Group. The PRECISE IPD Project Team will meet regularly every two to four months, usually by teleconference.

##### The PRECISE Trialist Group

Members of The PRECISE Trialist Group will be representatives of the eligible trials. For each trial, the first author will be invited to become a member of the Collaboration. If considered necessary or if there is no response from the first author, other investigators from the trials may be contacted (for example, the data manager or statistician). In order to keep The PRECISE Trialist Group updated, authors of new trials or previously unidentified trials will be contacted and invited to join the Collaboration in the course of the project.

##### The Data Management Group and PRECISE IPD Statistical Team

The data management group will be convened by the Chair of The PRECISE IPD Project Team and comprises statisticians from the data management centre and participating trials who will conduct the analyses. The data management group will be responsible for the storage and analyses of the IPD project data.

#### The PRECISE Group Meetings

Collaborative group face-to-face meetings will be organised at least twice during the study. Representatives of all eligible trials will be invited to attend those meetings. The meetings will be scheduled, if possible, in conjunction with international conferences. During those meetings, various aspects of the project will be discussed with all the collaborators, such as the project's design and conduct, the analysis plan and the interpretation and reporting of the results. The final collaborators' meeting is scheduled for 2012.

#### Publication of results

The final results of the study will be presented to the collaborators for discussion. The main manuscript will be prepared by The PRECISE IPD Project Team and Data Management Group and circulated to The PRECISE Group for comment and revision. The revised draft paper will be circulated for final comment and agreement prior to publication. PRECISE publications arising from these data will be authored with members of The PRECISE Group named where possible and on behalf of The PRECISE Collaboration as a whole. The names of all other collaborators participating will be acknowledged within the manuscript.

## Discussion

The recently updated meta-analysis [28] showed that the short term benefits seen for babies support the use of repeat dose(s) of prenatal corticosteroids for women who have received an initial course of prenatal corticosteroids seven of more days previously and who remain at risk of preterm birth.

For maximising benefit and minimising harm, many questions remain. Are repeat prenatal corticosteroids more effective in some women by reason of their risk of preterm birth? If the use of repeat prenatal corticosteroids is recommended, what is the best gestational age to use for benefit? What dose, number of repeat doses and timing prior to birth is optimal?

The best way to answer these remaining questions is to utilise existing IPD from all women and babies enrolled in previous trials of repeat prenatal corticosteroids. This approach has been described as the 'gold standard' of systematic review methodology as it allows for more powerful and flexible analysis of both subgroups and outcomes. The PRECISE Group has been formed to undertake a meta-analysis based on IPD, to answer these important clinical questions. Provision of data by the participating collaborators commenced in 2010 and results will be ready for presentation in 2012.

## Competing interests

The authors declare that they have no competing interests.

## Authors' contributions

CAC, FA, LMA, EVA, PB, TKB, LWD, SD, TJG, DAG, MH, MEH, PH, KM, PM, CM, PFM, KEM, OMP, DP, LS, EAT, MV, RJW, LY and SZ are all members of the PRECISE Study Group. The Chair of the PRECISE Project Team (CAC) wrote the first draft of the IPD protocol and prepared the initial draft of the manuscript. The PRECISE Project Team and The PRECISE Group participated in the protocol development, have commented on all drafts of the manuscript, and have read and approved the final draft of the manuscript.

## References

[B1] Australian & New Zealand Neonatal Network Report of the Australian and New Zealand Neonatal Network 2006 2009 Sydney: ANZNN

[B2] Kramer MS Demissie K Yang H Platt RW Sauve R Liston R The contribution of mild and moderate preterm birth to infant mortality JAMA 2000 284 7 843 849 10.1001/jama.284.7.843 10938173

[B3] Saigal S Doyle LW An overview of mortality and sequelae of preterm birth from infancy to adulthood Lancet 2008 371 9608 261 269 10.1016/S0140-6736(08)60136-1 18207020

[B4] Msall ME The panorama of cerebral palsy after very and extremely preterm birth: evidence and challenges Clin Perinatol 2006 33 2 269 270 10.1016/j.clp.2006.03.012 16765724

[B5] Doyle LW Victorian Infant Collaborative Study Group Evaluation of neonatal intensive care for extremely low birth weight infants in Victoria over two decades: II. Efficiency Pediatrics 2004 113 3 Pt 1 510 514 14993542 10.1542/peds.113.3.510

[B6] Doyle LW Victorian Infant Collaborative Study Group Changing availability of neonatal intensive care for extremely low birthweight infants in Victoria over two decades Med J Aust 2004 181 3 136 139 15287830 10.5694/j.1326-5377.2004.tb06203.x

[B7] Roberts D Dalziel S Antenatal corticosteroids for accelerating fetal lung maturation for women at risk of preterm birth Cochrane Database Syst Rev 2006 3 CD004454 16856047 10.1002/14651858.CD004454.pub2

[B8] Soll RF Morley CJ Prophylactic versus selective use of surfactant in preventing morbidity and mortality in preterm infants Cochrane Database Syst Rev 2000 2 CD000510 10796379 10.1002/14651858.CD000510

[B9] Crowther CA Harding JE Repeat doses of prenatal corticosteroids for women at risk of preterm birth for preventing neonatal respiratory disease Cochrane Database Syst Rev 2007 2 CD003935 17636741 10.1002/14651858.CD003935.pub2

[B10] McLaughlin KJ Crowther CA Walker N Harding JE Effects of a single course of corticosteroids given more than 7 days before birth: A systematic review Aust Nz J Obstet Gyn 2003 43 2 101 106 10.1046/j.0004-8666.2003.00052.x 14712961

[B11] McLaughlin KJ Crowther C Vigneswaran P Hancock E Willson K Who remains undelivered more than seven days after a single course of prenatal corticosteroids and gives birth at less than 34 weeks? Aust Nz J Obstet Gyn 2002 42 4 353 357 10.1111/j.0004-8666.2002.00353.x 12403279

[B12] Liggins GC Howie RN A controlled trial of antepartum glucocorticoid treatment for prevention of the respiratory distress syndrome in premature infants Pediatrics 1972 50 4 515 525 4561295

[B13] Quinlivan JA Evans SF Dunlop SA Beazley LD Newnham JP Use of corticosteroids by Australian obstetricians - a survey of clinical practice Aust Nz J Obstet Gyn 1998 38 1 1 7 10.1111/j.1479-828X.1998.tb02947.x 9521380

[B14] Brocklehurst P Gates S McKenzie-McHarg K Alfirevic Z Chamberlain G Are we prescribing multiple courses of antenatal corticosteroids? A survey of practice in the UK Brit J Obstet Gynaec 1999 106 9 977 979 10.1111/j.1471-0528.1999.tb08440.x 10492112

[B15] French NP Hagan R Evans SF Godfrey M Newnham JP Repeated antenatal corticosteroids: size at birth and subsequent development Am J Obstet Gynecol 1999 180 1 114 121 10.1016/S0002-9378(99)70160-2 9914589

[B16] Esplin MS Fausett M Smith S Oshiro B Porter T Branch D Varner M Multiple courses of antenatal steroids are associated with a delay in long-term psychomotor development in children with birth weight < 1500 grams Am J Obstet Gynecol 2000 182 1 part 2 S24

[B17] French NP Hagan R Evans SF Mullan A Newnham JP Repeated antenatal corticosteroids: effects on cerebral palsy and childhood behavior Am J Obstet Gynecol 2004 190 3 588 595 10.1016/j.ajog.2003.12.016 15041985

[B18] Aghajafari F Murphy K Ohlsson A Amankwah K Matthews S Hannah ME Multiple versus single courses of antenatal corticosteroids for preterm birth: a pilot study J Obstet Gynaecol Can 2002 24 4 321 329 12196868 10.1016/s1701-2163(16)30625-9

[B19] Crowther CA Haslam RR Hiller JE Doyle LW Robinson JS ACTORDS Study Group Neonatal respiratory distress syndrome after repeat exposure to antenatal corticosteroids: a randomised controlled trial Lancet 2006 367 9526 1913 1919 10.1016/S0140-6736(06)68846-6 16765760

[B20] Garite TJ Kurtzman J Maurel K Clark R Network OCR Impact of a 'rescue course' of antenatal corticosteroids: a multicenter randomized placebo-controlled trial Am J Obstet Gynecol 2009 200 3 248e1 9 19254583 10.1016/j.ajog.2009.01.021

[B21] Guinn DA Atkinson MW Sullivan L Lee M MacGregor S Parilla BV Davies J Hanlon-Lundberg K Simpson L Stone J Wing D Ogasawara K Muraskas J Single vs weekly courses of antenatal corticosteroids for women at risk of preterm delivery - a randomized controlled trial JAMA 2001 286 13 1581 1587 10.1001/jama.286.13.1581 11585480

[B22] Mazumder P Dutta S Kaur J Narang A Single versus multiple courses of antenatal betamethasone and neonatal outcome: A randomized controlled trial Indian Pediatr 2008 45 8 661 667 18723909

[B23] McEvoy C Bowling S Williamson K Lozano D Tolaymat L Izquierdo L Maher J Helfgott A The effect of a single remote course versus weekly courses of antenatal corticosteroids on functional residual capacity in preterm infants: a randomized trial Pediatrics 2002 110 2 280 284 10.1542/peds.110.2.280 12165579

[B24] Murphy KE Hannah ME Willan AR Hewson SA Ohlsson A Kelly EN Matthews SG Saigal S Asztalos E Ross S Delisle MF Amankwah K Guselle P Gafni A Lee SK Armson BA MACS Collaborative Group Multiple courses of antenatal corticosteroids for preterm birth (MACS): a randomised controlled trial Lancet 2008 372 9656 2143 2151 10.1016/S0140-6736(08)61929-7 19101390

[B25] Peltoniemi OM Kari MA Tammela O Lehtonen L Marttila R Halmesmaki E Jouppila P Hallman M Repeat Antenatal Betamethasone Study Group Randomized trial of a single repeat dose of prenatal betamethasone treatment in imminent preterm birth Pediatrics 2007 119 2 290 298 10.1542/peds.2006-1549 17272618

[B26] Wapner RJ Sorokin Y Thom EA Johnson F Dudley DJ Spong CY Peaceman AM Leveno KJ Harper M Caritis SN Miodovnik M Mercer B Thorp JM Moawad A O'Sullivan MJ Ramin S Carpenter MW Rouse DJ Sibai B Gabbe SG National Institute of Child Health and Human Development Maternal Fetal Medicine Units Network Single versus weekly courses of antenatal corticosteroids: evaluation of safety and efficacy Am J Obstet Gynecol 2006 195 3 633 642 10.1016/j.ajog.2006.03.087 16846587

[B27] McEvoy C Schilling D Peters D Tillotson C Spitale P Wallen L Segel S Bowling S Gravett M Durand M Respiratory compliance in preterm infants after a single rescue course of antenatal steroids: a randomized controlled trial Am J Obstet Gynecol 2010 202 6 544.e1 9 10.1016/j.ajog.2010.01.038 20227053 PMC2878893

[B28] Crowther CA McKinlay CJ Middleton P Harding JE Repeat doses of prenatal corticosteroids for women at risk of preterm birth for preventing neonatal health outcomes Cochrane Database Syst Rev 2011 6 CD003935 21678343 10.1002/14651858.CD003935.pub3 PMC4170912

[B29] Chalmers I The Cochrane collaboration: preparing, maintaining, and disseminating systematic reviews of the effects of health care Ann Ny Acad Sci 1993 703 156 165 10.1111/j.1749-6632.1993.tb26345.x 8192293

[B30] Stewart LA Tierney JF To IPD or not to IPD? Advantages and disadvantages of systematic reviews using individual patient data Eval Health Prof 2002 25 1 76 97 10.1177/0163278702025001006 11868447

[B31] Cochrane Collaboration IPD Group Higgins JPT, Green S Chapter 19 Reviews of individual patient data Cochrane Handbook for Systematic reviews of Interventions Version 500 2008 Chichester, UK: John Wiley & Sons Ltd [updated February 2008]

[B32] Stewart LA Clarke MJ Practical methodology of meta-analyses (overviews) using updated individual patient data. Cochrane Working Group Stat Med 1995 14 19 2057 2079 10.1002/sim.4780141902 8552887

[B33] Trial of the effects of antenatal multiple courses of steroids versus a single course (TEAMS): pilot study http://www.controlled-trials.com/ISRCTN46614711

[B34] Whitehead A Meta-analysis of controlled clinical trials 2002 Chichester, UK: John Wiley & Sons Ltd

[B35] Rothwell PM Treating individuals 2. Subgroup analysis in randomised controlled trials: importance, indications, and interpretation Lancet 2005 365 9454 176 186 10.1016/S0140-6736(05)17709-5 15639301

[B36] Roberts CL Lancaster PA Australian national birthweight percentiles by gestational age MJA 1999 170 114 118 10065122 10.5694/j.1326-5377.1999.tb127678.x

[B37] WHO Multicentre Growth Reference Study Group WHO Child Growth Standards based on length/height, weight and age Acta Paediatrica 2006 450 76 85 10.1111/j.1651-2227.2006.tb02378.x 16817681

